# Deep ocean carbonate ion increase during mid Miocene CO_2_ decline

**DOI:** 10.1038/srep04187

**Published:** 2014-02-26

**Authors:** Sev Kender, Jimin Yu, Victoria L. Peck

**Affiliations:** 1 British Geological Survey, Keyworth, Nottingham NG12 5GG, UK; 2 Department of Geology, University of Leicester, Leicester LE1 7RH, UK; 3 Research School of Earth Sciences, The Australian National University, Canberra, Australia; 4 British Antarctic Survey, High Cross, Madingley Road, Cambridge CB3 0ET, UK

## Abstract

Characterised by long term cooling and abrupt ice sheet expansion on Antarctica ~14 Ma ago, the mid Miocene marked the beginning of the modern ice-house world, yet there is still little consensus on its causes, in part because carbon cycle dynamics are not well constrained. In particular, changes in carbonate ion concentration ([CO_3_^2−^]) in the ocean, the largest carbon reservoir of the ocean-land-atmosphere system, are poorly resolved. We use benthic foraminiferal B/Ca ratios to reconstruct relative changes in [CO_3_^2−^] from the South Atlantic, East Pacific, and Southern Oceans. Our results suggest an increase of perhaps ~40 μmol/kg may have occurred between ~15 and 14 Ma in intermediate to deep waters in each basin. This long-term increase suggests elevated alkalinity input, perhaps from the Himalaya, rather than other shorter-term mechanisms such as ocean circulation or ecological changes, and may account for some of the proposed atmospheric CO_2_ decline before ~14 Ma.

The mid Miocene, including the mid Miocene climate transition (MMCT) at ~13.8 Ma^1^, represents one of the three most significant cooling episodes of the Cenozoic^2^,^3^, along with the Eocene-Oligocene transition^4^ (~34 Ma) and onset of Northern Hemisphere Glaciation (~2.7 Ma). Yet in contrast to these events, comparatively little is known about the causes and feedbacks of this mid Miocene cooling. Set within a long term cooling trend from 15 to 10 Ma, the rapid expansion of the East Antarctic ice sheet at 13.8 Ma^3^,^5^ is associated with a sea level fall in the order of ~60 m^6^,^7^, and a coeval drop in deep ocean temperature of ~2°C^2^,^8^. This glacial expansion was accompanied by the extinction of Antarctic tundra^9^, a ~6–7°C fall in sea surface temperature in the high latitude southwest Pacific^10^, the development of perennial sea ice in the Arctic^11^,^12^, the development of significant temperate terrestrial biotic provinces including the early expansion of C_4_ grasses^13^, and significant tectonic events that would have impacted on the global carbon cycle (see Supplementary Information). Hypotheses for the causes of this transition include CO_2_ drawdown, perhaps from a cessation of mid Miocene volcanism^14^ or enhanced terrestrial weathering and erosion^15^, priming the climate system for ice sheet growth during low amplitude orbital eccentricity at 13.8 Ma^1^. Alternatively the onset of a deep Antarctic Circumpolar Current may have thermally isolated Antarctica due to tectonic subsidence in the Scotia Sea^16^.

There have been numerous efforts to reconstruct atmospheric CO_2_ through the Miocene^14^,^17^,^18^,^19^, and in contrast to previous suggestions that temperature may have become decoupled from CO_2_ during the mid Miocene^17^,^18^, a recent study suggests a decline of ~100 ppm may have occurred between ~15 and 14 Ma before the MMCT^14^. As surface ocean CO_2_ is in approximate steady state with the atmosphere, and the relatively small surface ocean reservoir is continuously replenished by the much larger deep ocean reservoir (>1 km), the speciation of dissolved inorganic carbon (DIC) of the deep ocean is linked to atmospheric CO_2_ (refs 20,21) and therefore a ~100 ppm decrease in CO_2_ should be documented within deep sea sediments. In the ocean, DIC occurs as several species that maintain acid/base equilibrium: 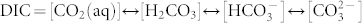


As the ratio of ocean water alkalinity (ALK; the sum of bases in solution, in sea water predominantly: [HCO_3_^−^] + 2[CO_3_^2−^] + [B(OH)_4_^−^]) to DIC increases (for example from increased continental weathering or increased biological productivity), the proportion of the DIC pool present as sea water [CO_3_^2−^] increases, thus shifting the balance away from [CO_2_(aq)] and potentially drawing down atmospheric CO_2_ to recover equilibrium^20^,^21^. Previous attempts to constrain past ocean carbonate chemistry have focused on reconstructions of the calcite compensation depth (CCD) linked to [CO_3_^2−^], which are of low resolution and subject to significant complications^22^, or reconstructions of [CO_3_^2−^] directly with benthic foraminiferal shell Li/Ca and Mg/Ca^7^, also subject to complications such as a temperature effect^7^ (Supplementary Information).

Recent studies^23^,^24^ on core top samples from several ocean basins have shown a strong quantitative relationship between benthic foraminiferal B/Ca ratios and deep water [CO_3_^2−^], which has been further supported by subsequent down-core studies^25^,^26^,^27^. Here, we reconstruct deep and intermediate water [CO_3_^2−^] from six Ocean Drilling Program (ODP) core sites in three ocean basins (Fig. 1) using B/Ca of the epifaunal benthic foraminifera *Cibicidoides mundulus* (Fig. 2, see Methods). The species *C. mundulus* is unique in being long-ranging, allowing the use of this proxy as far back as the mid Miocene.

## Results

### *Cibicidoides mundulus* B/Ca

In the ~1 Ma lead up to the MMCT (~15.5–14 Ma), benthic foraminiferal B/Ca ratios from all six sites increase (Fig. 2, Supplementary Table S1), indicating a probable increase in deep water [CO_3_^2−^]. All samples have low Al/Ca values well below 100 μmol/mol (Supplementary Table S1), indicating little potential clay contamination^7^,^24^. In the South Atlantic (Fig. 2b) the deepest Site 1226 (~3.6 km palaeo-water depth) shows a larger increase in B/Ca over this interval than shallower Site 1264 (2.3 km), indicating a possible greater increase in [CO_3_^2−^] in deep waters relative to intermediate waters. In the East Pacific (Fig. 2d) and Southern Ocean (Fig. 2e), the magnitude of the B/Ca increase at both the shallow and deep sites appears to be comparable, with a slightly larger overall increase in the East Pacific perhaps due to a modified ocean circulation pattern. In the ~1 Ma after the MMCT (~14–13 Ma) deeper sites in the East Pacific (Site 1237, 2.8 km) and Southern Ocean (Site 1168, 2.3 km) exhibit further increased benthic B/Ca, whilst at nearby shallower Sites 1236 (0.9 km) and 1171 (2 km) benthic B/Ca is seen to drop slightly. This B/Ca decrease at the shallower sites may be explained by either a decrease in global intermediate water [CO_3_^2−^] perhaps due to changes in ocean circulation, or more likely, localised depletions in [CO_3_^2−^] due to expansion of the oxygen minimum zones associated with enhanced primary productivity (both sites are situated in proximity to the modern depth of [CO_3_^2−^] and oxygen minima; Supplementary Fig. S1).

### Miocene oceanic [CO_3_^2−^]

B/Ca ratios from all sites were converted to [CO_3_^2−^] using the 0.69 μmol/mol per μmol/kg sensitivity obtained from a global core-top calibration^23^, past water depth estimates, and mid Miocene estimates of seawater boron and calcium ratios (B/Ca_sw_) and concentrations (Supplementary Information). There is yet to be a consensus on mid Miocene boron and calcium concentrations^28^,^29^ and future estimates may therefore change, although the relative changes in [CO_3_^2−^] inferred from our records (Fig. 3) may not be greatly affected. It is likely that the B/Ca_sw_ ratio changed over the interval 15.5–13 Ma, even considering the long residence times of boron (20 Ma)^30^ and calcium (1.1 Ma)^31^ in the oceans. It is difficult to estimate these potential changes, but the dominant source of oceanic boron is considered to be continental discharge from weathering of silicates, carbonates and evaporites^32^, and changing Himalayan erosion has been identified as an important variable on Cenozoic boron flux^28^. To test the sensitivity of our [CO_3_^2−^] estimates to a changing flux of boron to the oceans, we consider the effect of a 10% increase in B/Ca_sw_ given that sedimentation rates from Asian basins are estimated to have increased by ~10% due to Himalayan erosion between 15.5 and 13 Ma^33^ (Supplementary Fig. S2). A 10% increase in oceanic boron whilst calcium remained stable (conservatively assuming the majority of weathering products were silicates^34^), would have increased B/Ca_sw_ by ~3 μmol/mol, impacting [CO_3_^2−^] estimates by only ~4 μmol/kg (included in the error on Fig. 3). Calcium concentrations of the oceans between 15.5–13 Ma are actually estimated to have increased in the order of 50%^29^, largely from exposure of shelf carbonates as sea level fell particularly after ~13.8 Ma^29^. Since the B/Ca of the exposed carbonates is unknown, the impact on B/Ca_sw_ is unknown. However if calcium concentrations drove B/Ca changes, then we would expect to see similar changes in Mg/Ca and Li/Ca (Supplementary Table S1), which we do not. With the exception of the two data points from the shallowest sites (Sites 1236 and 1171) showing a decrease at ~13.5 Ma, we estimate deep water [CO_3_^2−^] to have increased by an average of perhaps ~40 μmol/kg between ~15.5 and 13 Ma, with the largest increase occurring between ~15 and 14 Ma (Fig. 3d).

**Figure 1:**
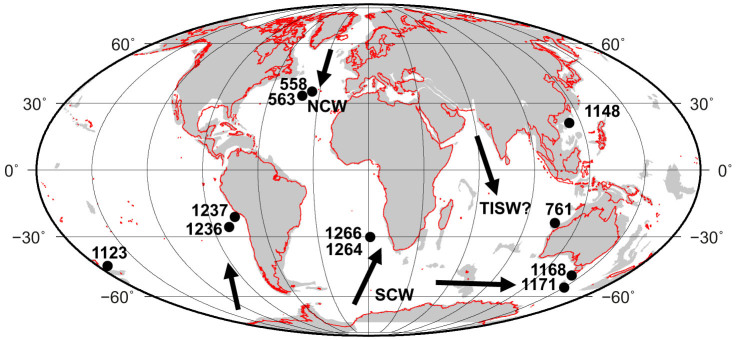
Global palaeogeographic map showing the continental configuration at 14 Ma. The positions of ODP Sites discussed in this study are indicated as filled circles. Possible major sources of deep Northern Component Water (NCW), Southern Component Water (SCW), and Tethys-Indian Saline Water (TISW), are indicated by schematic arrows. Mollweid projection of modern continents (red) is shown on a palaeogeographic reconstruction, generated from^52^, of continental plates (grey) centered at 14 Ma.

**Figure 2:**
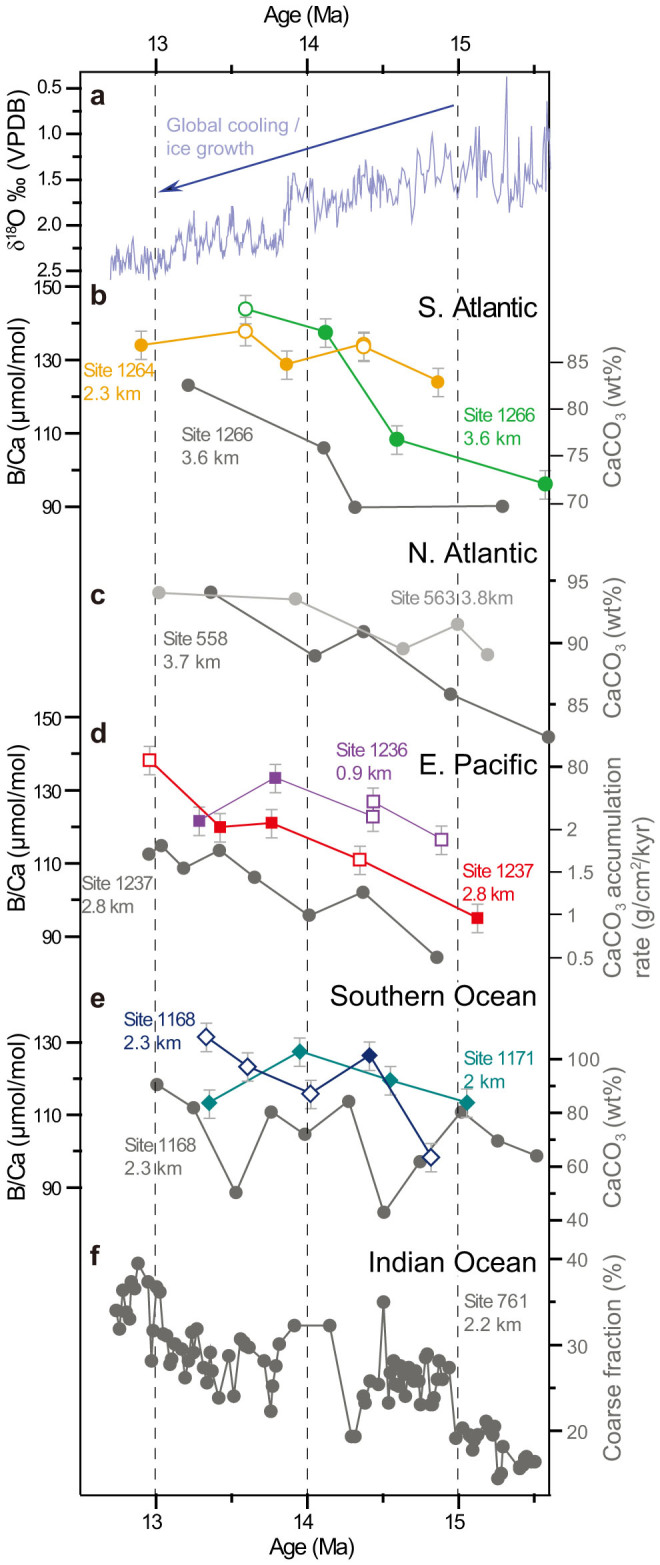
Summary of ocean carbonate proxies. Deep-ocean B/Ca records (coloured symbols) compared with deep-ocean sedimentary CaCO_3_ data (grey symbols) and deep-ocean oxygen isotopes (blue line) over the interval ~15 to 13 Ma. Open symbols indicate probable interglacial samples. Increasing values of B/Ca and CaCO_3_ data indicates elevated bottom water [CO_3_^2−^]. (a) Deep ocean δ^18^O from Site 1237^3^, indicating long term cooling with the sharpest drop at ~13.8 Ma during the mid Miocene climate transition. (b) B/Ca with CaCO_3_ wt% data from the Walvis Ridge^53^. (c) CaCO_3_ wt% data from the northern Mid-Atlantic Ridge^54^. (d) B/Ca with CaCO_3_ accumulation rate data from the Nazca Ridge^3^,^55^. (e) B/Ca with CaCO_3_ wt% data from the Tasmanian Margin and South Tasman Rise^56^. (f) CaCO_3_ coarse fraction data from the Wombat Plateau^7^.

**Figure 3:**
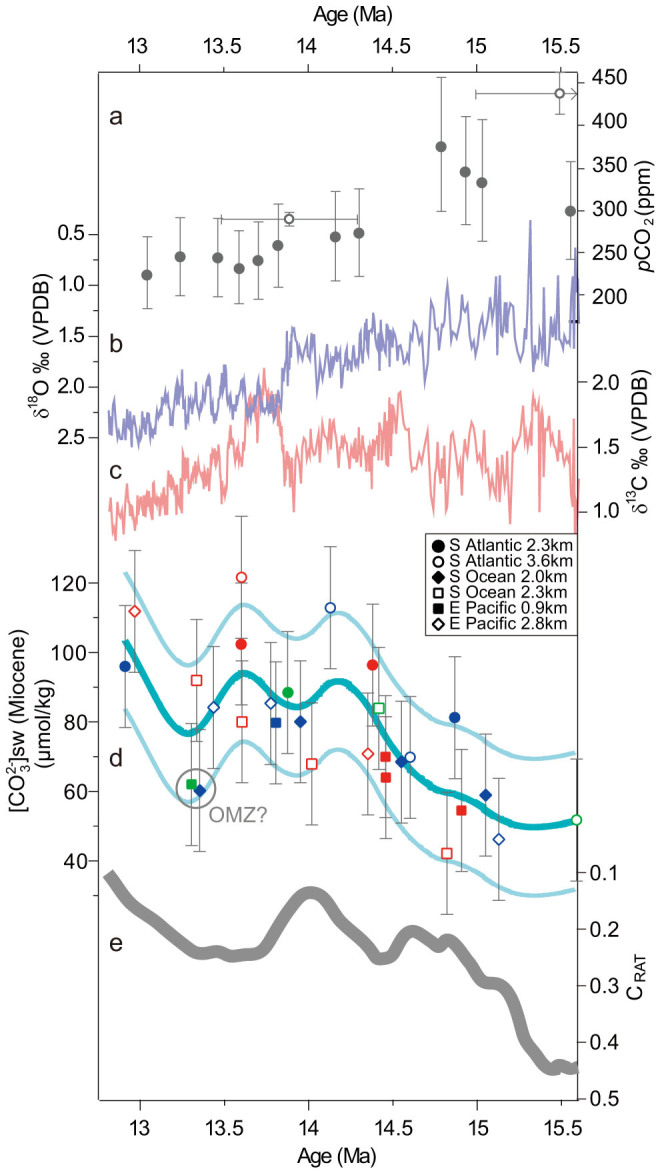
Composite seawater [CO_3_^2−^] estimates against other mid Miocene climate records. (a) Atmospheric CO_2_ reconstructed from planktonic foraminiferal δ^11^B (filled circles)^14^, and fossil leave stomatal frequency (open circles)^19^. (b) Deep ocean benthic foraminiferal δ^18^O from ODP Site 1237^3^, indicating long term global cooling and ice-sheet expansion during the mid Miocene, and rapid cooling and ice sheet expansion at ~13.8 Ma. (c) Deep ocean δ^13^C from Site 1237^3^. (d) Deep-ocean [CO_3_^2−^] estimated from foraminiferal B/Ca data of several sites (Fig. 2), using estimates for mid Miocene B/Ca_sw_ values and calculated palaeo-water depths (see Methods). Error largely associated with calibration uncertainty^23^ and possible changes to B/Ca_sw_ due to continental weathering (see Methods). Blue symbols represent glacials, red interglacials, and green intermediate. Blue line is a best fit 5-point smoothing spline, with ±1s.d. of dataset (light blue). Circle indicates two samples that may have been affected by local oxygen minimum zone (OMZ) expansion. (e) Chemical weathering index from ODP Site 1148, South China Sea, as the ratio of chlorite/(chlorite + haematite + goethite) (C_RAT_)^33^. Lower values may represent increasing monsoon intensity over Southern China and associated intense weathering of the Himalaya^33^. Other proxies (see Supplementary Information) indicate increased Himalayan weather and erosion during the mid Miocene.

Over the interval ~15.5–13 Ma, deep water CaCO_3_ preservation may have increased in the South Atlantic, East Pacific and Southern Ocean (Fig. 2b, d and e), possibly reflecting the increasing [CO_3_^2−^] (from B/Ca) and inferred deepening of the CCD. Carbonate preservation proxies in the North Atlantic (Fig. 2c, wt% CaCO_3_), Indian Ocean (Fig. 2f, % CaCO_3_ coarse fraction), West Pacific (Supplementary Fig. S3, CaCO_3_ fragmentation) and Southwest Pacific (Supplementary Fig. S3, wt% CaCO_3_) show a long term increase, suggesting possible increases in [CO_3_^2−^] in these locations also. There are significant uncertainties with using these records to infer preservation, as we do not yet have detailed sedimentation rate or CaCO_3_ rain rate data^22^, but taken together the CaCO_3_ trends are fairly consistent. The consistency of our [CO_3_^2−^] reconstruction and CaCO_3_ preservation records (Figs 2,3d) appears to point to a global increase in [CO_3_^2−^] of deep (>1 km) waters particularly from ~15–14 Ma, as our records cover a large depth range (~0.9–3.8 km) and geographic distribution of the major ocean basins (Fig. 1), and include both glacial and interglacial measurements (Fig. 3d). A study from ODP Site 761 at 2.2 km water depth in the Indian Ocean also concluded that there was an increase in local [CO_3_^2−^] between ~15 and 14 Ma^7^.

We recognise that there is substantial variability within our dataset, partly because the measurements come from various sites and from various times corresponding to both glacial and interglacial intervals and oceanic [CO_3_^2−^] may be expected to have decreased during interglacials^26^. However the long term increase in [CO_3_^2−^] from ~15 to 14 Ma presented here is superimposed onto these shorter time-scale variations. Significant basin partitioning of CaCO_3_ may not be expected since similar values of benthic δ^13^C in both the Pacific and Atlantic Oceans between 15 and 12 Ma (Supplementary Fig. S4) suggests a relatively homogenous deep water mass may have been present in the Southern Ocean^3^ and possibly the South Atlantic. Modelling experiments suggest that ocean circulation was dominated by Southern Ocean deep water mass formation^35^, and sedimentological proxies document increasing influence of a deep Pacific southern water mass after ~15 Ma^36^. Miocene Tethys Indian Saline Water, a significant component of deep and intermediate water bathing the Indian and Pacific Oceans (Fig. 1) and possibly warming Antarctica, appears to have diminished by ~15 Ma^37^ likely due to the shoaling of the Tethys Ocean basin^37^. Northern Component Water is not thought to have played a prominent role in ocean circulation until after ~12 Ma^35^,^38^, possibly due to the shoaling of the Greenland-Scotland Ridge. Neodymium isotopes from Walvis Ridge (used as an ocean circulation tracer) show no increased stratification and therefore North Atlantic deep water formation before ~10.7 Ma^39^.

## Discussion

A long-term, whole ocean increase in [CO_3_^2−^] at >1 km water depth and apparently enhanced CaCO_3_ preservation (Fig. 2), particularly apparent between ~15 and 14 Ma (Fig. 3d), implies an increase in the whole ocean ratio of alkalinity to dissolved inorganic carbon (ALK:DIC), as a charge imbalance driven by ALK is balanced by the speciation of DIC away from [CO_2_] towards [CO_3_^2−^] (ref. 21). ALK is largely supplied to the ocean by continental weathering of emergent carbonates and silicates on land, and removed from the ocean by burial of CaCO_3_ on the sea floor^20^. DIC is added and removed by these processes, but also removed by burial of organic carbon (C_org_) in ocean sediments, and by air-sea exchange. There are several ways of causing increases in deep ocean [CO_3_^2−^], including a lowering of the CaCO_3_/C_org_ rain ratio of primary export production^20^, increased shelf to basin fractionation from sea level fall and exposure and erosion of carbonate shelves^4^,^20^,^26^,^27^,^40^, changes in ocean circulation^20^,^26^, increased high latitude nutrient utilization increasing the [CO_3_^2−^] of preformed surface water^20^,^41^, and increased continental weathering^15^,^20^,^33^,^42^. Processes such as ocean circulation and nutrient utilization can produce large short-term excursions in seawater [CO_3_^2−^], but are less likely to account for the long-term change observed from ~15.5 to 13 Ma. This is because carbonate compensation, the process by which an elevation in deep water [CO_3_^2−^] increases the area of CaCO_3_ burial on the sea floor and the subsequent removal of ocean water [CO_3_^2−^], would bring the ocean to the initial steady-state on a timescale of several thousand years^43^.

Increased shelf to basin fractionation was proposed as the major cause of the widespread CCD fall during ice sheet expansion at the Eocene-Oligocene transition, as sea level fall drove carbonate deposition deeper into basins and raised oceanic δ^13^C^4^,^40^. However there is no evidence for a long-term sea level fall^6^ or long term δ^13^C increase (Fig. 3c) prior to 14 Ma, when we infer a notable [CO_3_^2−^] increase (Fig. 3d). If a large, long-term decrease in the CaCO_3_/C_org_ ratio were to account for some of the [CO_3_^2−^] variability from ~15.5–13 Ma, as has been suggested to account for the short-term δ^13^C maxima at ~13.7 Ma^44^, we would expect to see increasingly organic-rich sediments in marine sediment cores. In fact, the interval ~16–14 Ma is associated with episodic increases in organic rich sediments around the Pacific margin^45^ that are associated with global δ^13^C and δ^18^O maxima (Fig. 3), and higher productivity along the west coast of Africa^46^ before ~14 Ma, indicating that changes in the CaCO_3_/C_org_ ratio may account for part the observed changes in [CO_3_^2−^] particularly before 14 Ma. However, no long-term secular increase in δ^13^C, consistent with a sizeable increase in productivity, is apparent in any records (Fig. 3c, Supplementary Fig. S4).

We believe that the most plausible explanation for the long-term increase in [CO_3_^2−^] suggested by our records, particularly before 14 Ma, was increased continental weathering and erosion^15^. Uplift and erosion of the Himalaya, which has been linked to Cenozoic climate change via the erosion and weathering of silicates which consumes CO_2_ (refs 33,34), would also have added ALK to the ocean. The Greater Himalaya experienced enhanced exhumation rates during the mid Miocene^33^ (Supplementary Fig. S2c), and plate configuration reconstructions indicate the Himalaya met with the Intertropical Convergence Zone of high precipitation in the mid Miocene^42^. Several mineralogical proxies associated with monsoonal intensity also point to an increase in Himalayan regional chemical weathering in the mid Miocene^33^ (Fig. 3e). Mounting evidence in support for enhanced weathering rates, and ultimately delivery of ALK to the oceans, is consistent with a peak in bulk sediment accumulation rates in the major clastic basins around Southeast Asia^33^ (supplied by the Himalaya), and high fluxes of Sr from the Ganges-Brahmaputra Rivers^47^ during the mid Miocene (Supplementary Fig. S2e). Although dating is not yet able to precisely constrain the timings of all these events, if enhanced silicate weathering and erosional deposition from the Himalaya was responsible for global cooling during the mid Miocene, as has been previously suggested^15^,^33^,^42^, then an increase in ocean ALK and hence [CO_3_^2−^] would be expected to be coeval. Other possible terrestrial sources of ALK were the Tethys Ocean, which underwent uplift and intermittent closure in the mid Miocene^48^, and the East Africa Plateau which also experienced peak uplift in the mid Miocene^49^ (Supplementary Fig. S2c), although the timing and magnitude of these events are even less well constrained. Whichever the source, enhanced continental weathering, consuming atmospheric CO_2_ and transporting ALK to the ocean, is consistent with our interpretation of elevated seawater [CO_3_^2−^] during the mid Miocene.

In summary, our new reconstruction of deep ocean [CO_3_^2−^] (>1 km water depth) provides a tantalising insight into potential changes in the carbonate system during the mid Miocene. Comparison of these records with published δ^18^O, δ^13^C, and CO_2_ proxy records (Fig. 3, Supplementary Fig. S5) indicates enhanced input of ALK to the oceans as a possible contributor to mid Miocene climate change before the MMCT at 13.8 Ma. A likely source could have been the Himalaya which experienced increased uplift, weathering and erosion centred at ~15–14 Ma (Supplementary Fig. S2). Whilst our records demonstrate that carbonate chemistry in the world's oceans changed during the mid Miocene, they also highlight the need to better resolve deep and intermediate water [CO_3_^2−^] to fully understand the roles of weathering, ocean circulation and the carbon cycle during this complex episode of climatic change. However, taking our data at face value, we performed simple sensitivity calculations of the effect a hypothetical addition of ALK to achieve the estimated [CO_3_^2−^] increase of 40 μmol/kg (Fig. 3d) may have had on ocean water CO_2_, using modelled estimates of initial Miocene ocean water ALK, DIC and temperature (Supplementary Information). Although necessarily subject to significant uncertainties such that absolute values must be considered speculative, if the deep ocean [CO_3_^2−^] increase was mixed to surface water, it may have drawn down atmospheric CO_2_ in the region of ~16–25% (Supplementary Table S2). This is broadly in the range of atmospheric CO_2_ reconstructions from fossil leaf stomatal frequency^19^ and planktonic foraminiferal δ^11^B (Fig. 3a)^14^. Further, in agreement with δ^11^B reconstructions, the timing of our increase in deep ocean [CO_3_^2−^] and putative drawdown in atmospheric CO_2_ is consistent with the largest drawdown in CO_2_ taking place between ~15 and 14 Ma before the abrupt MMCT at 13.8 Ma^14^, and comparison of these two records does indicate a possible coupling between atmospheric CO_2_ and deep ocean [CO_3_^2−^] (Supplementary Fig. S5). Atmospheric CO_2_ may have also been affected by other mechanisms such as volcanism^14^,^47^, and our reconstructions contain too much uncertainty to indicate how much CO_2_ was removed by the oceans. However, the general increase in deep ocean [CO_3_^2−^] over the mid Miocene presented here does indicate a drawdown of atmospheric CO_2_ into the ocean may have occurred, and highlights the importance of constraining deep ocean changes when assessing mid Miocene carbon cycle dynamics.

## Methods

### Analytical procedures

Deep sea sediment samples of ~40 cc volume were washed through a 63 μm sieve with deionised water, and oven dried at <30°C. A total of 10–25 specimens of the benthic foraminifera *Cibicidoides mundulus* were picked from the 250–350 μm fraction in each sample, and cleaned using the standard ‘oxidative’ treatment protocol^50^,^51^. Elemental/Ca ratios were analyzed by inductively coupled plasma mass spectrometer (ICP-MS) according to methods described elsewhere^23^,^51^. For the ICP-MS, the B blank is <2% of the consistency standard (B/Ca = 150 μmol/mol). Recent detailed work on the foraminiferal genus *Cibicidoides* indicates that different morphospecies produce a certain degree of variation in B/Ca (ref. 24). To minimise possible error, we strictly selected only ‘typical’ *C. mundulus* using a conservative species concept (see Supplementary Information), from the 250–350 μm size fraction, and a relatively high number of specimens (>20 individuals where available). Replicate analysis carried out on two samples showed variability of <4 μmol/mol, in line with published replicate analyses of standards and samples giving precisions of <2% (RSD) for B/Ca^23^.

### Miocene [CO_3_^2−^] estimates

Values for Δ[CO_3_^2−^] were calculated by modifying the equation: *B/Ca = aΔ[CO_3_^2−^] + b* (ref. 23), where *a* = 0.69 and *b* = 119.1. To estimate mid Miocene B/Ca_sw_ values (modern B = 415 μmol/kg; modern Ca = 0.01 mol/kg), we assumed B to be ~1.07 times the modern value^28^, and Ca to be ~1.12 times the modern value^29^. The resulting value for Miocene B/Ca_sw_ is ~96% of modern, which was used to modify the equation: B/Ca_(Miocene)_ = 0.96 * aΔ[CO_3_^2−^] + 0.96 * b. The calculated Δ[CO_3_^2−^] was converted to [CO_3_^2−^] by adding the [CO_3_^2−^]_sat_ of the pressure equivalent to estimated water depths at ~14 Ma. See Supplementary Information for further details.

### Assessment of uncertainty

The error bars associated with B/Ca values (Fig. 2) include an analytical precision of ±1.5 μmol/mol, and a replicate error of ±2 μmol/mol. The error bars associated with [CO_3_^2−^]_sw_ (Fig. 3d) include the analytical precision of ±2.2 μmol/kg, the replicate error of ±2.9 μmol/kg, a calibration uncertainty of ±10 μmol/kg (2 σ)^23^, a sea level uncertainty of ±0.5 μmol/kg (from ~50 m change), and a mid Miocene ΔB/Ca_sw_ uncertainty (from increased terrestrial erosion) of ±2 μmol/kg.

## Author Contributions

S.K., V.L.P. and J.Y. conceived and designed the project, J.Y., S.K. and V.L.P. generated data and S.K., J.Y. and V.L.P. wrote the paper.

## Supplementary Material

Supplementary Information Supplementary Information - KENDER

Supplementary Information Supplementary Tables 1 and 2 KENDER
