# Supplementary material for: Deep ocean carbonate ion increase during mid Miocene CO2 decline
Source: Sci Rep. 2014 Feb 26;4:4187. doi: 10.1038/srep04187 (PMC3935200; doi:10.1038/srep04187)
Supplement: Supplementary Information — KENDER [file srep04187-s1.pdf]

SUPPLEMENTARY INFORMATION

Deep ocean carbonate ion increase during mid Miocene CO_2 decline

Sev Kender[1,2], Jimin Yu[3] and Victoria L. Peck[4]

[1]British Geological Survey, Keyworth, Nottingham NG12 5GG, UK

[2]Department of Geology, University of Leicester, Leicester LE1 7RH, UK

[3]Research School of Earth Sciences, The Australian National University, Canberra, Australia

[4]British Antarctic Survey, High Cross, Madingley Road, Cambridge CB3 0ET, UK

Possible [CO_3^{2-}] effect on CO_2. Increased oceanic [CO_3^{2-}] driven by ALK input from enhanced weathering would absorb atmospheric CO_2 into surface ocean water[1]. Partitioning of [CO_3^{2-}] between deep and surface waters is linked to the strength and mode of overturning circulation. Shifting $\delta^{13}C$ gradients suggest that the loss of CO_2 from the deep ocean during the last glacial cycle was related to a change in the mode of oceanic circulation[2]. The absence of any mid Miocene change in the $\delta^{13}C$ gradient between deep and shallow Pacific records (e.g. ODP Sites 1237, 1146 and 588)[3] does not indicate such a long term change in Pacific deep water [CO_3^{2-}] partitioning occurred. However, our South Atlantic records (Fig. 2b) do indicate a greater increase in [CO_3^{2-}] at the deeper site (3.6 km) than the shallower site (2.3 km) from ~14.5 Ma. It is likely that surface ocean [CO_3^{2-}] was at least partially modified by deep water [CO_3^{2-}] through overturning and mixing[4] , as the time interval our records cover (10^6 yr) would allow for complete mixing (modern ocean mixing time is 10^3 yr). The deep ocean increase in [CO_3^{2-}] of ~40 µmol/kg from ~15–13 Ma ago (Fig. 3d) would have therefore been at least partially transferred to surface waters which would have acted to lower surface water and atmospheric CO_2 partial pressure (pCO_2) (ref. 1).

In order to estimate the effect a surface water increase in [CO_3^{2-}] of ~40 µmol/kg would have had on atmospheric CO_2, we performed sensitivity calculations on a range of different ocean parameters (Supplementary Table S2) assuming the increase in deep water [CO_3^{2-}] (Fig. 3d) was transferred to surface waters, and that surface waters were in approximate equilibrium with the atmosphere during the mid Miocene. We use initial ALK and DIC from two modelled data sets: ALK:DIC of 1600 and 1400 µmol/kg respectively[5] and ALK:DIC at 1900 and 1700 µmol/kg respectively[6]. Salinity was assumed similar to modern as CO_2 and [CO_3^{2-}] has been shown to be relatively insensitive to salinity[5], and our predicted changes in pCO_2 are comparatively insensitive to the initial selection range of temperature and salinity conditions. We increase ocean [CO_3^{2-}] by inputting ALK:DIC at various ratios. Sensitivity calculations indicated that a ±1 ‰ change in resultant salinity affected $\%\Delta pCO_2$ by ±2%, and so salinity was left unchanged for the calculations shown in Supplementary Table S2. Silicate weathering (largely Ca and Mg silicates from the

Himalaya[7]) adds ALK:DIC to the ocean in a 2:2 ratio ($CaSiO_3 + 2CO_2 + 3H_2O \rightarrow Ca^{2+} + 2HCO_3^- + H_4SiO_4 \rightarrow CaCO_3 + SiO_2 + CO_2 + 3H_2O$), but uses 2 moles of carbon from the atmosphere. This carbon is returned to the atmosphere, as the ocean/atmosphere act as a single reservoir on a timescale of several thousand years, and so the net addition of ALK:DIC to the ocean from silicate weathering is 2:0. Carbonate weathering adds ALK:DIC to the ocean in a 2:1 ratio. The increase in oceanic ALK:DIC causes absorption of CO_2 from the atmosphere, which adds ALK:DIC to the ocean in a 0:1 ratio. Although a lowering of the CCD increases $CaCO_3$ burial and acts to lower ocean ALK:DIC in a 2:1 ratio, our results (Figs 2,3) show the integrated response of ocean water to the total addition and removal. The effect that sulfide and evaporite weathering would have had on ALK:DIC is not taken into account here as they form a comparatively minor contribution (silicates alone probably accounted for over 80% of Neogene Himalayan weathering products[7]), although for more accurate total ALK:DIC estimates this deserves further investigation.

When changing ALK:DIC at 2:1±0.3 (Supplementary Table S2), an increase in surface water $[CO_3^{2-}]$ of ~40 µmol/kg would have drawn down pCO_2 by ~65 to 100 ppm for an initial ALK:DIC of 1900 and 1700 µmol/kg, or ~45 to 80 ppm for an initial ALK:DIC of 1600 and 1400 µmol/kg. Whatever the initial conditions, a consistent decrease in pCO_2 of ~16–25 % was found, depending on the ratio of ALK:DIC added but independent of initial ALK:DIC values, in line with atmospheric CO_2 reconstructions from fossil leaf stomatal frequency[8] and planktonic foraminiferal $\delta^{11}B$ (ref. 9).

Comparison of Li/Ca, Mg/Ca and B/Ca proxies. A previous study reconstructed benthic $[CO_3^{2-}]$ during the mid Miocene at ODP Site 761 (Fig. 1) using combined Mg/Ca and Li/Ca ratios of the benthic foraminifera *C. mundulus* and *Oridorsalis umbonatus*[10]. As Mg/Ca and Li/Ca are sensitive to both temperature and $\Delta[CO_3^{2-}]$, a combined equation was derived to reconstruct temperature and $\Delta[CO_3^{2-}]$. Applying the equation to our Mg/Ca and Li/Ca data collected in the same samples as B/Ca (apart from Site 1264, as it may have been outside of the Mg/Ca saturation state effect due to higher $\Delta[CO_3^{2-}]$; Supplementary Fig. S1), we find no meaningful correlation between reconstructed $\Delta[CO_3^{2-}]$ from Li/Ca and Mg/Ca and reconstructed $\Delta[CO_3^{2-}]$ from B/Ca (Supplementary Fig. S7), and conclude that at our sites the Li/Ca and Mg/Ca method may not be suitable to reconstruct $\Delta[CO_3^{2-}]$.

Supplementary Methods

Age models. Published age models were used for Sites 1264[11], 1237[3], 1236[12], 1168[13], 1171[14], 558 and 563[15] based varyingly on biostratigraphy, magnetostratigraphy, isotope stratigraphy, and cyclostratigraphy. For Site 1266 we inferred a linear sedimentation rate between the first occurrence of *Triquetrorhabdulus rugosus*[16] dated at 13.28 Ma[17], and the first occurrence of *Sphenolithus heteromorphus*[16] dated at 17.71 Ma[17]. The $\delta^{18}O$ increase of ~0.6‰ at ~13.8 Ma (Supplementary Fig. S6) confirms the position of the MMCT.

$CaCO_3$ records. $CaCO_3$ mass accumulation rate (MAR) was calculated for Site 1237 using the equation: $CaCO_3$ MAR ($g/cm^2/kyr$) = linear sedimentation rate (cm/kyr) x sediment dry density (g/cm^3) x $CaCO_3$ (dry weight fraction). Linear sedimentation rates were taken from[3], and dry density and %$CaCO_3$ was taken from[18]. At Sites 1266[16], 558[19], 563[19] and 1168[20], wt% $CaCO_3$ was used due to the lower resolution age models that do not allow changes in sedimentation rate between ~12 and 16 Ma to be resolved. At Site 761[10], % carbonate coarse fraction was used as there was no wt% $CaCO_3$, and more corrosive water is thought to fragment the coarse fraction.

Miocene water depths. In order to convert sea water $\Delta[CO_3^{2-}]$ (calculated using the equation below[21]) to $[CO_3^{2-}]$, it is necessary to know $[CO_3^{2-}]_{sat}$ which is a function of pressure (water depth). The water depth at ~14 Ma was calculated for Sites 1168, 1171, 1236, 1237, 1264 and 1266 by using estimates of the underlying oceanic crust age[11,12,22] and assuming standard subsidence rates over the last 14 Ma[23]. As Sites 1168 and 1171 are situated over subsided continental crust, we estimated mid Miocene water depths by using the age of the proximal oceanic crust at ~34 Ma[22]. Our depth estimates of 2140 m and 1780 m for Sites 1168 and 1171 respectively are in line with micropalaeontological and sedimentological constraints which indicate the sites were greater than 1500 m depth at ~14 Ma[22], and similar to a previous estimate of 1600 m for Site 1171[14]. Although these estimates are necessarily subject to error, this error is of limited importance for our purposes as a difference in water depth of ~300 m only affects calculated sea water $[CO_3^{2-}]$ values by ~6%. Although absolute $[CO_3^{2-}]$ values may change slightly with more accurate water depth estimates in the future, the trend in sea water $[CO_3^{2-}]$ over the interval 15–13 Ma (Figs 2,3) should be unaffected by the absolute water depth.

Species selection. For trace metal analysis we selected the epifaunal benthic foraminifera *Cibicidoides mundulus* (Brady, Parker, and Jones, 1888), as its shell B/Ca values have a high sensitivity to changes in benthic $[CO_3^{2-}]$ (refs 21,24). Recent studies have highlighted the importance of selecting this species *sensu stricto* when attempting to reconstruct seawater $[CO_3^{2-}]$, as other species that have some morphological similarities to *C. mundulus* could yield variable values[24]. In this study we use specimens that are between 250–350 µm in size, and which conform to the species description given by Holbourn & Henderson[25]. In particular *C. mundulus* contains 10-12 chambers in the final whorl, which increase in size only gradually as added. This differs from *Planulina wuellerstorfi* (Schwager, 1866), and intergradations between the two species, which have more rapidly increasing chambers in the final whorl[24,25] and were excluded from analysis.

Miocene [CO_3^{2-}] estimate. In order to account for mid Miocene sea water B/Ca values that were different from modern, we assumed B concentration to be ~1.07 modern value[26] and Ca concentration to be ~1.12 modern value[27].

Modern calibration: $B/Ca = a * \Delta[CO_3^{2-}] + b$,
where a = 0.69, b = 119.1
Miocene calibration: $B/Ca = r * a * \Delta[CO_3^{2-}] + r * b$,
where: $r = (B/Ca^{Miocene}) / (B/Ca^{modern})$

Modern values: [B] = 415 μmol/kg, [Ca] = 0.01 mol/kg, B/Ca_{sw} = 41 mmol/mol.
Miocene (~14 Ma): $[B]_{Miocene}$ = ~1.07 * modern [B]; $[Ca]_{Miocene}$ = ~1.12 * modern [Ca]; thus, $B/Ca_{Miocene} = 0.96 * B/Ca_{sw}^{modern}$, average: r = 0.96 of modern B/Ca.

Supplementary References

1. Zeebe, R. E. & Wolf-Gladrow, D. *CO_2 in Seawater: Equilibrium, Kinetics, Isotopes* (Elsevier Oceanography Series, Amsterdam, 2004).
2. Yu J. M. *et al*. Loss of carbon from the deep sea since the Last Glacial Maximum, *Science* **330,** 1084–1087 (2010).
3. Holbourn, A., Kuhnt, W., Schultz, M., Flores, J-A. & Anderson N. Orbitally-paced climate evolution diring the middle Miocene “Monterey” carbon-isotope excursion. *Earth Planet. Sci. Lett.* **261,** 534–550 (2007)**.**
4. Sigman, D. M. & Boyle, E. A. Glacial/interglacial variations in atmospheric carbon dioxide. *Nature* **407,** 859–869 (2000).
5. Tyrell, T. & Zeebe, R. E. History of carbonate ion concentration over the last 100 million years. *Geochem. et Cosmochem. Acta* **68,** 3521–3530 (2004).
6. Berner, R. A. & Kothavala, Z. GEOCARB III: A revised model of atmospheric CO2 over phanerozoic time. *American J. of Sci.* **301,** 182–204 (2001).
7. France-Lanord, C. & Derry, L. A. Organic carbon burial forcing of the carbon cycle from Himalayan erosion *Nature* **390,** 65–67 (1997).
8. Kürschner, W. M., Kvaček, Z. & Dilcher, D. L. The impact of Miocene atmospheric carbon dioxide fluctuations on climate and the evolution of terrestrial ecosystems. *Proc. Natl. Acad. Sci. USA* **105,** 449–453 (2008).
9. Foster, G. L., Lear, C. H. & Rae, J. W. B. The evolution of pCO_2, ice volume and climate during the middle Miocene. *Earth Planet. Sci. Lett.* **341–344,** 243–254 (2012).

10. Lear, C. H., Mawbey, E. M. & Rosenthal, Y. Cenozoic benthic foraminiferal Mg/Ca and Li/Ca records: towards unlocking temperatures and saturation states. *Paleoceanography* **25,** PA4215, doi:10.1029/2009PA001880 (2010).
11. Shipboard Scientific Party, Leg 208 summary. *Proc. Ocean Drill. Program Init. Reports* **208,** 1–112 (2004).
12. Shipboard Scientific Party, Leg 202 summary. *Proc. Ocean Drill. Program Init. Reports* **202,** 1–145 (2003).
13. Stickley C. E. *et al.* Late Cretaceous–Quaternary biomagnetostratigraphy of ODP Sites 1168, 1170, 1171, and 1172, Tasmanian Gateway. *Proc. Ocean Drill. Program Sci. Results* **189,** 1–57 (2004).
14. Shevenell, A. E., Kennett, J. P. & Lea, D. W. Middle Miocene Southern Ocean cooling and Antarctic cryosphere expansion. *Science* **305,** 1766–1770 (2004).
15. Miller, K. G. & Fairbanks, R. G. Oligocene to Miocene global carbon isotope cycles and abyssal circulation changes. *In* Sundquist, E. T. & Broecker, W. S. (eds.). *The Carbon Cycle and Atmospheric* CO_2*: Natural Variations Archean to Present* pp. 469–486 (American Geophysical Union, Washington, DC, 1985).
16. Shipboard Scientific Party, Site 1266. *Proc. Ocean Drill. Program Init. Reports* **208,** 1–79 (2004).
17. Gradstein, F. M. & Ogg, J. G. *A Geologic Time Scale 2004* (Cambridge University Press, Cambridge, 2005).
18. Shipboard Scientific Party, Site 1237. *Proc. Ocean Drill. Program Init. Reports* **202,** 1–107 (2003).
19. Bougault H. *et al., Initial Reports of the Deep Sea Drilling Project* (U.S. Government Printing Office, Washington, DC, 1985).
20. Shipboard Scientific Party, Site 1168. *Proc. Ocean Drill. Program Init. Reports* **189,** (2001). Available from: World Wide Web: <http://www-odp.tamu.edu/publications/189_IR/chap_03/chap_03.htm>.
21. Yu, J. M. & Elderfield, H. Benthic foraminiferal B/Ca ratios reflect deep water carbonate saturation state. *Earth Planet. Sci. Lett.* **258,** 73–86 (2007).
22. Exon, N. F., Kennett, & J. P. Malone, M. J. Leg 189 synthesis: Cretaceous–Holocene history of the Tasmanian Gateway. *Proc. Ocean Drill. Program Sci. Results* **189,** 1–21 (2004).
23. Sclater, J. G., Meinke, L., Bennett, A. & Murphy, C. The depth of the ocean through the Neogene. Kennett, J. P. (ed.). *In The Miocene Ocean: Paleoceanography and Biogeograph*y pp. 1–21 (The Geological Society of America, Boulder, CO, 1985).
24. Rae, J. W. B., Foster, G. L., Schmidt, D. N. & Elliott, T. Boron isotopes and B/Ca in benthic foraminifera: proxies for the deep ocean carbonate system. *Earth Planet. Sci. Lett.* **302,** 403–413 (2011).

25. Holbourn, A. E. & Henderson, A. S. Re-illustration and revised taxonomy for selected deep-sea benthic foraminifers. *Palaeontol. Electron.* **4(2),** 34pp (2002), http://palaeo-electronica.org/paleo/2001_2/foram/issue2_01.htm
26. Lemarchand, D., Gaillardet, J., Lewin, É. & Allégre, C. J. Boron isotope systematics in large rivers: implications for the marine boron budget and paleo-pH reconstruction over the Cenozoic. *Chem. Geol.* **190,** 123–140 (2002).
27. Griffith, E. M., Paytan, A., Caldeira, K., Bullen, T. D. & Thomas, E. A dynamic marine calcium cycle during the past 28 million years. *Science* **322,** 1671–1674 (2008).
28. Schlitzer, R. World Ocean Circulation Experiment results, http://www.ewoce.org/ (accessed August 2013).
29. Clift, P. D. *et al*. Correlation of Himalayan exhumation rates and Asian monsoon intensity. *Nature Geosci.* **1,** 875–880 (2008).
30. Wichura, H., Bousquet, R., Oberhänsli, R., Strecker, M. R. & Trauth, M. H. Evidence for middle Miocene uplift of the East African Plateau. *Geology* **38,** 543–546 (2010).
31. Rögl, F. Mediterranean and Paratethys. Facts and hypotheses of an Oligocene to Miocene paleogeography (short overview). *Geol. Carpathica* **50,** 339–349 (1999).
32. Derry, L. A. & France-Lanord, C. Neogene Himalayan weathering history and river $^{87}Sr/^{86}Sr$: impact on the marine Sr record. *Earth Planet. Sci. Lett.* **142,** 59–74 (1996).
33. Tian J. *et al*. Reorganization of Pacific Deep Waters linked to middle Miocene Antarctic cryosphere expansion: A perspective from the South China Sea. *Palaeogeogr. Palaeoclimatol. Palaeoecol.* **284,** 375–382 (2009).
34. Hall, I. R. *et al.* Paleocurrent reconstruction of the deep Pacific inflow during the middle Miocene: Reflections of East Antarctic Ice Sheet growth. *Paleoceanography* **18,** 1040, doi:10.1029/2002PA000817 (2003).
35. Zachos, J. C., Pagani, M., Sloan, L., Thomas, E. & Billups, K. Trends, Rhythms, and Aberrations in Global Climate 65 Ma to Present. *Science* **292,** 686–693 (2001).
36. Lear, C. H., Elderfield, H. & Wilson, P. A. Cenozoic deep-sea temperatures and global ice volumes from Mg/Ca in benthic foraminiferal calcite. *Science* **287,** 269–272 (2000).
37. Pelletier, G., Lewis, E. & Wallace, D. *A calculator for the CO2 system in seawater for Microsoft Excel/VBA.* (Washington State Department of Ecology, Olympia, WA, Brookhaven National Laboratory, Upton, NY, ed. 1.0, 2005).

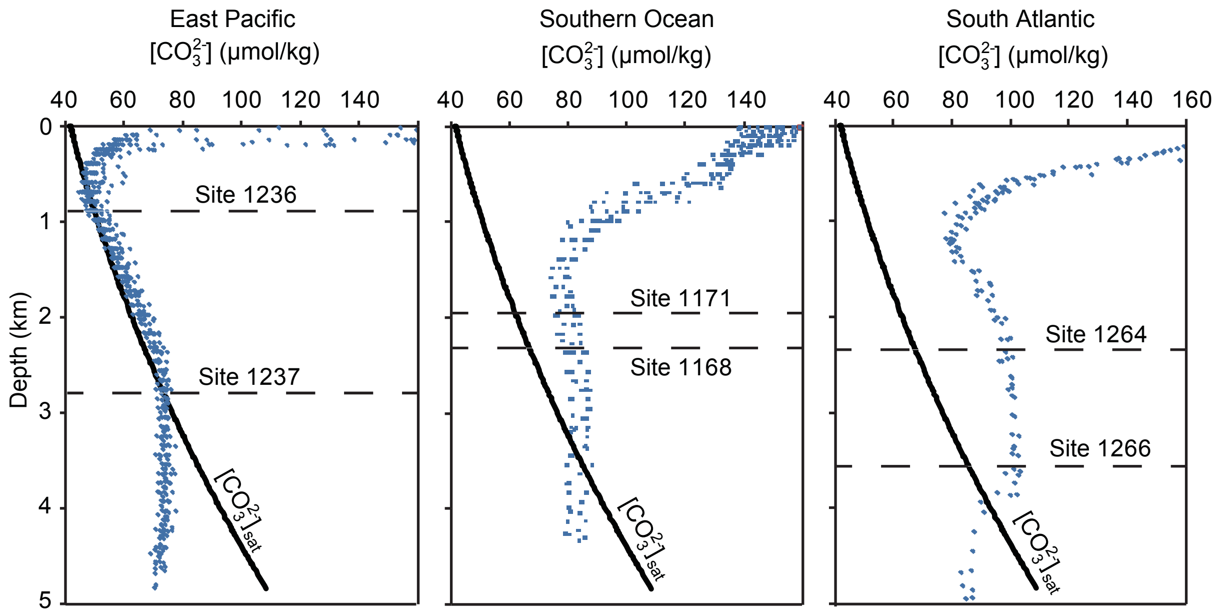

Figure S1. Modern $[CO_3^{2-}]$ profiles for the sites in this study, with $[CO_3^{2-}]_{sat}$ values. The $[CO_3^{2-}]$ data is taken from the World Ocean Circulation Experiment results[28].

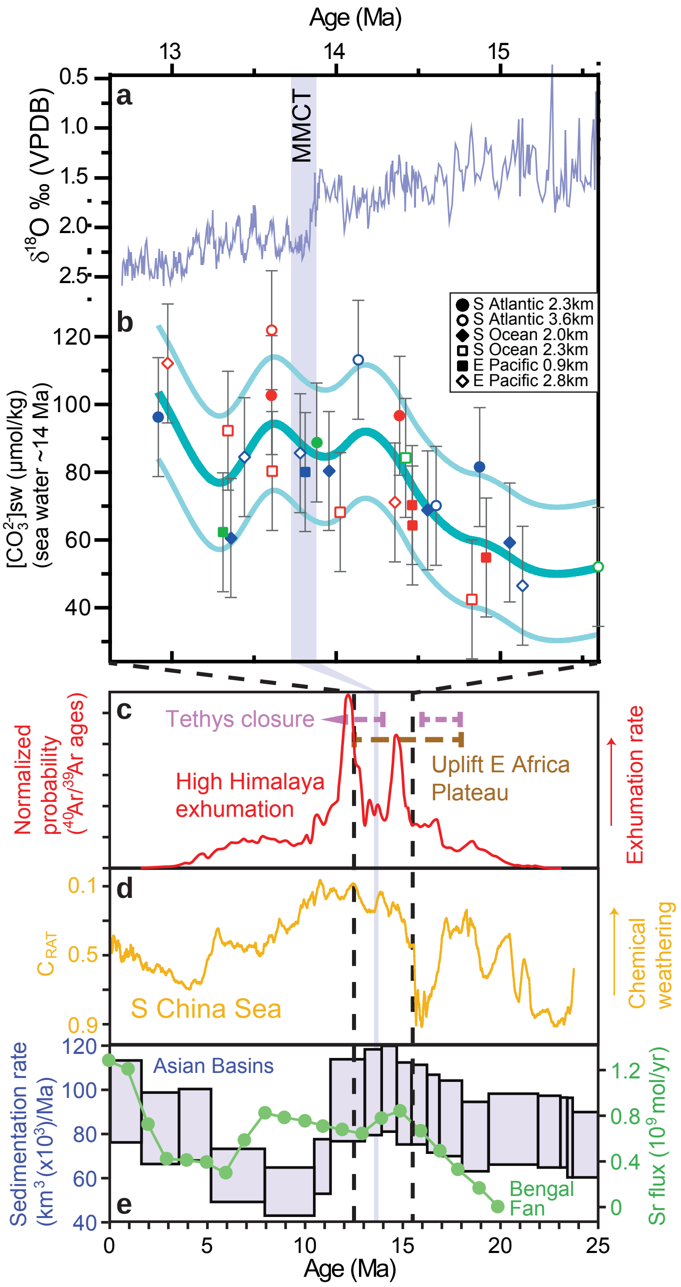

Figure S2. Summary of relevant tectonic-related information over the interval 25 to 0 Ma. (a) Deep ocean $\delta^{18}O$ from Site 1237[3], indicating global cooling and ice-sheet expansion during the mid Miocene climate transition. (b) Deep-ocean $[CO_3^{2-}]$ calculated from foraminiferal B/Ca data of several sites (Fig. 2), using estimates for mid Miocene B/Ca_{sw} values and calculated palaeo-water depths (see Methods). Error largely associated with calibration uncertainty[21] and possible changes to B/Ca_{sw} due to continental weathering (see Methods). Blue symbols represent glacials, red interglacials, and green intermediate. Blue line is a best fit 5-point smoothing spline, with ±1s.d. of dataset (light blue). (c) 3142 white mica $^{40}Ar/^{39}Ar$ ages from the High Himalaya[29], representing mica cooling supposedly during exhumation. Higher values represent a higher frequency of dates and therefore greater bedrock erosion[29]. Also shown are the times of peak East African Plateau uplift and erosion (brown dashed line[30]), and uplift and closure of the Tethys Ocean (purple dashed line[31]), which may also have added alkalinity to the ocean. (d) Chemical weathering index from ODP Site 1148, South China Sea, as the ratio of chlorite / (chlorite + haematite + goethite) (C_{RAT}) (ref. 29). Lower values may represent increasing monsoon intensity over Southern China[29]. (e) Integrated budget of sediment accumulation rates from major Asian basins[29] (Mekong, Gulf of Thailand, South China, Red River and Indus Fan), indicating a peak in Himalayan erosion during the mid Miocene. Modelled flux of Sr from the Ganges and Brahmaputra Rivers[32], from Bengal Fan ODP Sites 717 and 718 $^{87}Sr/^{86}Sr$ records and global seawater $^{87}Sr/^{86}Sr$ records. Higher values indicate elevated weathering of the Himalaya.

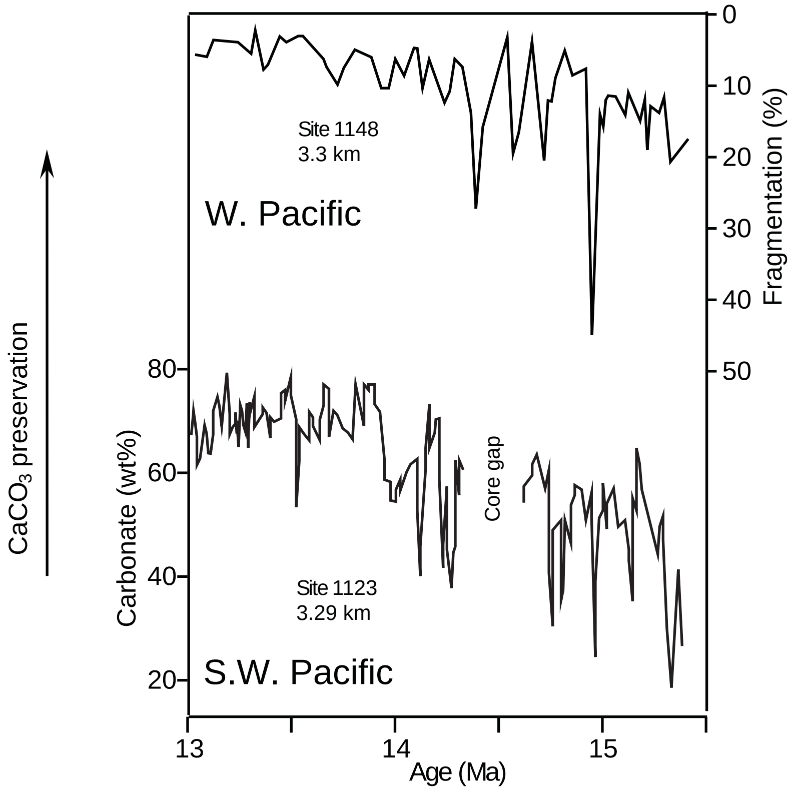

Figure S3. Fragmentation % of West Pacific ODP Site 1148 foraminifera shells[33], used as a proxy for dissolution, and wt% $CaCO_3$ of Southwest Pacific ODP Site 1123[34].

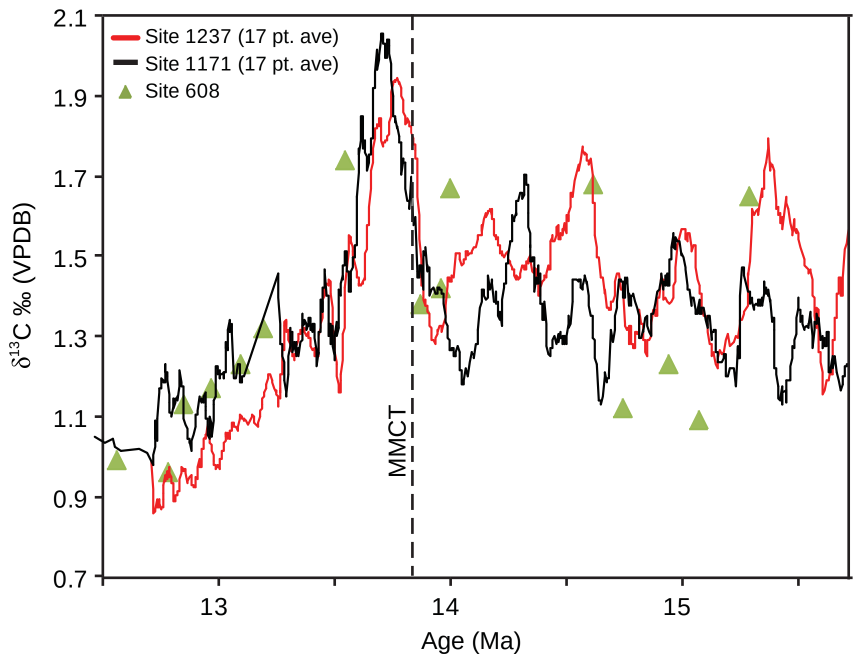

Figure S4. Benthic $\delta^{13}C$ from ODP Sites 1237 (S.E. Pacific[3]), 1171 (Southern Ocean[14]) and 608 (North Atlantic[35]). Note there is no significant change in the offsets between these records, indicating no major changes in ocean basin partitioning over this interval.

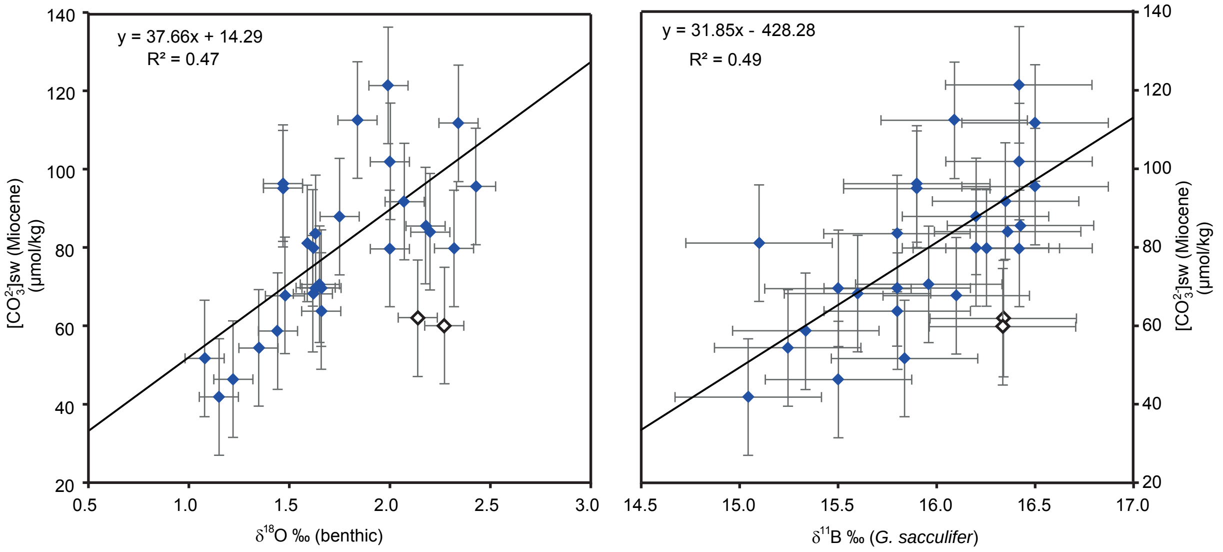

Figure S5. Comparison of our reconstructed mid Miocene seawater $[CO_3^{2-}]_{sw}$ with (a) deep ocean benthic $\delta^{18}O$ from Site 1237[3], used to estimate the combined effect of global glaciation and temperature, and (b) planktonic foraminiferal $\delta^{11}B$ (ref. 9) from Site 761, used to calculate atmospheric CO_2. Where actual $\delta^{11}B$ measurements were not available for certain time intervals of $[CO_3^{2-}]_{sw}$ values, corresponding values for $\delta^{11}B$ were calculated using a linear interpolation between measured points[9]. Symbols in white indicate two samples that may have been affected by local oxygen minimum zone expansion (see Results), and were therefore not included in the calculation of the linear regression. Uncertainty for the $[CO_3^{2-}]_{sw}$ is largely associated with calibration uncertainty[21] and possible changes to B/Ca_{sw} due to continental weathering (see Methods). Uncertainty for $\delta^{18}O$ and $\delta^{11}B$ is the sum of analytical uncertainty[9].

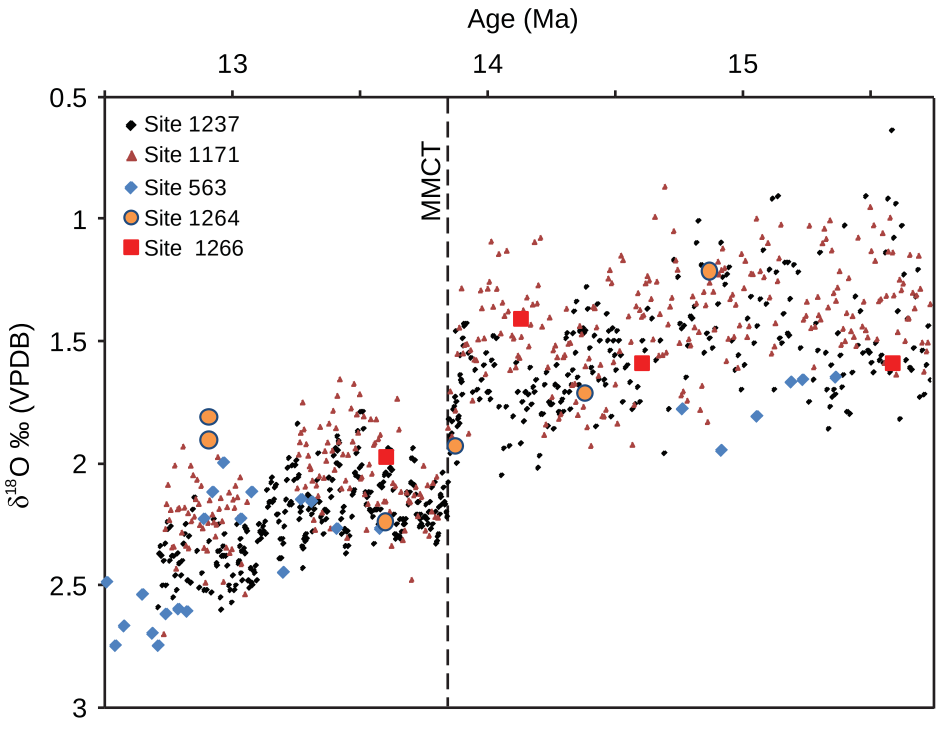

Figure S6. Benthic $\delta^{18}O$ from ODP Sites 1237 (S.E. Pacific[3]), 1171 (Southern Ocean[14]), 563 (North Atlantic[35]), and 1264 and 1266 (South Atlantic). All records contain the significant $\delta^{18}O$ increase over the mid Miocene climate transition (MMCT) at ~13.8 Ma.

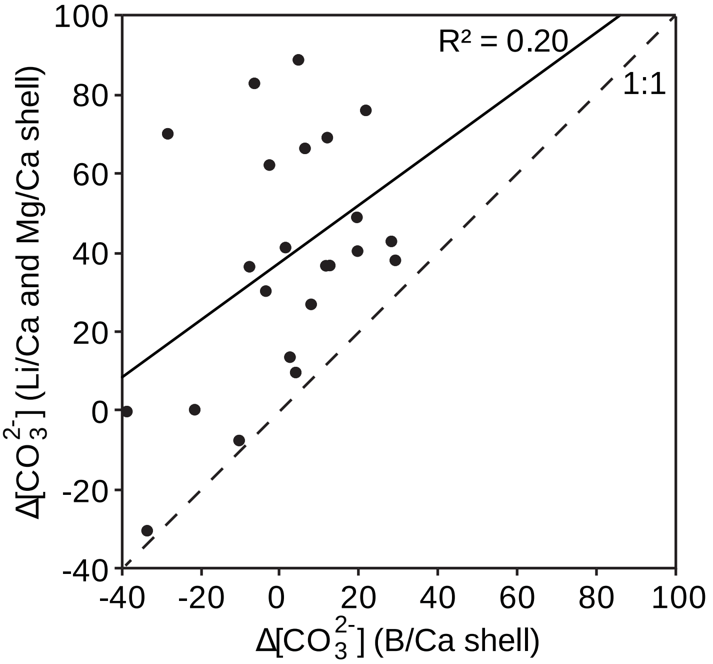

Figure S7. Values of ocean water $\Delta[CO_3^{2-}]$ calculated from *C. mundulus* B/Ca (see Methods), against ocean water $\Delta[CO_3^{2-}]$ calculated from *C. mundulus* Li/Ca and Mg/Ca according to the equation of[10]. These data do not include Site 1264, which may have been outside of a Mg/Ca saturation state effect due to higher $\Delta[CO_3^{2-}]$ (ref. 10).

Supplementary Table Captions

Table S1. Mg/Ca, Al/Ca, Li/Ca and B/Ca results from all Ocean Drilling Program (ODP) samples, together with sample depth and age information, and calculated palaeo-water depths and $[CO_3^{2-}]$ values. $\Delta[CO_3^{2-}]$ and $[CO_3^{2-}]$ were calculated using the equation of[21]. $\Delta[CO_3^{2-}]_{Miocene}$ and $[CO_3^{2-}]_{Miocene}$ were calculated with an adjusted equation taking into account differences in sea water B and Ca at ~14 Ma (see discussion above).

Table S2. Changes in pCO_2 after the addition of alkalinity (ALK) and dissolved inorganic carbon (DIC) to the ocean at different ratios, from different starting values. Initial ALK and DIC of 1600 and 1400 µmol/kg respectively is from[5], and 1900 and 1700 µmol/kg from[6]. ALK and DIC was added to the surface ocean at fixed ratios to achieve an increase in surface ocean $[CO_3^{2-}]$ of ~40 µmol/kg. Average global temperature (T) was taken as 6±1°C warmer than modern[36], and salinity was assumed similar to modern (35 ‰) due to its minor effect on CO_2 and $[CO_3^{2-}]$ results[5]. CO_2sys.xls (version 14)[37] was used for calculations, with the following parameters selected: pH on the seawater scale; K1 and K2 equilibrium constants from Mehrbach et al.; KSO_4 equilibrium constant from Dickson; pressure 2800 dbar for deep ocean, 0 for surface; phosphate 2.3 µmol/kg for deep ocean, 0 for surface; silicate 110 µmol/kg for deep ocean, 0 for surface; temperature 2°C for deep ocean, 21°C for surface.
